# Diagnosis and Classification of Pediatric Epilepsy in Sub-Saharan Africa: A Comprehensive Review

**DOI:** 10.3390/jcm13216396

**Published:** 2024-10-25

**Authors:** Sofia Di Noia, Linda Bonezzi, Ilaria Accorinti, Emanuele Bartolini

**Affiliations:** 1 Neuropediatric Unit, Woman and Child Department, Polyclinic of Foggia, 71122 Foggia, Italy; sofiadinoianpi@gmail.com; 2 Tuscany PhD Programme in Neurosciences, 50139 Florence, Italy; 3 School of Medicine, Faculty of Medicine and Biomedical Sciences, The University of Queensland, Brisbane 4072, Australia; linda.bonezzi@fsm.unipi.it; 4 Department of Developmental Neuroscience, IRCCS Stella Maris Foundation, 56128 Pisa, Italy; ilaria.accorinti@fsm.unipi.it; 5 Department of Clinical and Experimental Medicine, University of Pisa, 56126 Pisa, Italy

**Keywords:** childhood epilepsy, LMIC, pediatric, epidemiology, challenges

## Abstract

**Background/Objectives**: Epilepsy is a major public health issue in Sub-Saharan Africa, particularly among children, due to limited healthcare resources, socioeconomic inequalities, and cultural stigma that often result in underdiagnosis and undertreatment. This review examines pediatric epilepsy’s diagnosis, classification, and management in this setting, highlighting the need for culturally appropriate interventions to improve care quality and address these challenges. **Methods**: A review of the literature was conducted using MEDLINE, Embase, Scopus, and Web of Science databases to identify pertinent studies published between 2013 and 2024. This review included studies examining the epidemiology, seizure classification and etiologies of epilepsy among children in Sub-Saharan Africa. **Results**: This review revealed higher incidence and prevalence of epilepsy in Sub-Saharan Africa compared to high-income countries, primarily attributable to factors such as infectious diseases, perinatal injuries, and limited diagnostic resources. The most frequently reported types of epilepsy were generalized and focal seizures, with significant etiological contributions from structural and infectious causes, including nodding syndrome and HIV-related epilepsy. The treatment gap remains considerable, with up to 80% of children not receiving appropriate antiseizure medications. **Conclusions**: The diagnosis and treatment of epilepsy in pediatric populations in Sub-Saharan Africa is complicated by several factors, including cultural stigma and the lack of adequate healthcare infrastructure. There is an urgent need for culturally tailored diagnostic tools, improved access to affordable treatments, and public health initiatives aimed at reducing stigma. Addressing these gaps through enhanced research, improved healthcare access, and targeted educational campaigns is crucial for improving the quality of life for children with epilepsy.

## 1. Introduction

Epilepsy is a neurological disorder characterized by the recurrence of unprovoked seizures and by the associated neurobiological, cognitive, psychological, and social consequences [1]. It constitutes a major public health challenge worldwide.

The World Health Organization (WHO) estimates that about 5 million people are diagnosed with epilepsy each year. Global lifetime prevalence is higher than 50 million, making epilepsy the second most common neurological disorder after stroke. Nearly 80% of people with epilepsy (PWE) live in low- and middle-income countries (LAMICs), where the epidemiological and social burden of epilepsy is significantly higher [2,3]. The underlying etiology is variable—spanning from genetic, brain structural, infectious, metabolic, immune, or unknown causes—and depends on the geographical location. In high-income countries (HICs), acquired brain lesions (e.g., stroke, brain tumors) play a major role, whilst in LAMICs, infectious disorders and traumatic brain injuries are prevailing [4].

To diagnose epilepsy and to ascertain the etiology can be difficult tasks, especially in LAMICs, where socioeconomic and cultural constraints can be obstacles to the recognition and acceptance of the disease.

PWE must face a wide array of hurdles for wellbeing, both related to the largely unpredictable nature of seizures themselves and also to the adverse events of medications, social limitations, public stigma, psychiatric, cognitive, and somatic comorbidities. These factors often hamper a satisfiable quality of life and can be especially dooming for children. At the pediatric age, coping strategies to prevent social isolation and discrimination are frailer. Mental health and psychological wellbeing can be neglected in geographical regions affected by economic difficulties or faraway from metropolitan centers such as many African regions, especially in rural areas.

Indeed, epilepsy can have a devastating impact on individuals and families, leading to health, social, psychological, and economic hardship. Furthermore, epilepsy affects not only the wellbeing of the individual but also influences resource allocation for health systems that have to manage a wide number of critical conditions.

Epilepsy carries a significant impact on disability-adjusted life years (DALYs)—a measure of years lost due to ill health, disability, or early death [5]. In 2016, epilepsy was associated with more than 13 million DALYs. While this figure appears in a steady reduction trend for HICs, LAMICs still struggle with an unchanged health and socioeconomic impact [5,6,7,8].

Such complex background mandates us to enhance the understanding of the specific challenges for PWE in Africa.

Although the International League Against Epilepsy (ILAE) Pediatric Commission Research Advocacy Task Force has recently recognized a remarkable increase in epilepsy research capability in Africa over the past 30 years, there remains a significant need to strengthen the region’s capacity, both in terms of study quality and broader coverage of relevant areas [9].

In the current review, we focus on the available evidence on the epidemiology and clinical characterization of epilepsy, the subjective experiences of patients and caregivers, the challenges and barriers to care, the cultural factors that need to be considered when developing interventions, and the best practices for providing epilepsy care in Africa. Our purpose is to provide a state-of-the-art scenario to raise awareness on the topic, with special attention to consider culturally appropriate interventions.

We aim particularly to describe the specific challenges in diagnosis and classification that are fundamental to establish the correct treatment and management of children with epilepsy in Sub-Saharan Africa.

## 2. Materials and Methods

The current review was conducted according to the Preferred Reporting Items for Systematic Reviews and Meta-Analyses statement using the extension checklist for scoping reviews (PRISMA-ScR) [10].

To identify relevant papers, we systematically searched MEDLINE (accessed by PubMed), Embase, Scopus and Publons/Web of Science databases for full-text articles published from 2013 up to 8 August 2024, using the following search terms: epidemiol* AND africa* AND child* AND epilep*.

Using automated filters, we excluded (i) abstract and conference papers and (ii) studies published in language other than English. We then manually assessed the titles and abstracts of the retrieved papers to exclude those unrelated to the topic of interest. Endnote web reference manager was used to exclude duplicate papers. The full text of all potentially eligible articles and their Supplementary Information were independently assessed by two authors (S.D.N. and E.B.) and summarized through joint discussion. In the final analysis, we included 11 studies with focus on seizure classification, 14 studies on epilepsy epidemiology, and 11 studies on epilepsy etiology.

## 3. Results

### 3.1. Epidemiology: Incidence and Prevalence

Most incidence studies have been conducted in HICs, often with a suboptimal design such as a retrospective observation rather than community-based surveys. Nevertheless, available data from Africa have steadily increased and are not to be neglected. Incidence studies are important for identifying risk factors as well as for providing information on prognosis, assessing the number of new cases of epilepsy in a well-defined population during a specified period (e.g., 1 year or lifetime).

In a systematic review and meta-analysis study, Fiest et al. calculated the incidence rate of epilepsy to be 61.4 per 100,000 person-years (95% CI 50.7–74.4), with higher values in LAMICs compared to HICs (139 vs. 48.9 per 100,000 person-years) [11] (Table 1). Such difference highlights the different composition of the population at risk as well as the heterogeneity of causative factors [11]. In HICs, the incidence follows a typical U-shape, with peaks in early childhood and in the elderly. Conversely, in LAMICs the epilepsy incidence is definitely higher in early childhood and progressively decreases over time. In Sub-Saharan Africa (SSA), the vast majority of people with incident epilepsy are younger than 20 years old, and the elderly-age peak is absent [12]. This may be due to the lower life expectancy, with consequent less exposure to the typical factors promoting elderly-onset epilepsy in HICs (e.g., cerebrovascular and neurocognitive diseases), in addition to the increased mortality rate associated with epilepsy and its underlying causes. Perinatal causes such as birth asphyxia are a powerful driver of seizures in Africa that may also affect mortality [13]. Of note, Fiest et al. found that cumulative incidence was not different worldwide; the rare occurrence of elderly-onset epilepsy in LAMICs appears to be counterbalanced by a higher peak in childhood [11].

However, the epidemiological view of epilepsy according to socioeconomic and geographical location can be blurred by sampling biases. The incidence might be overestimated in world regions characterized by limited diagnostic resources and less stringent epidemiological criteria [4]. With this regard, studies conducted in resource-poor countries are heterogeneous and partially conflicting. The incidence reported in Uganda [14] is twice that of Tanzania [15] and of Ethiopia [16], which have figures comparable to those of Western countries. Local etiological factors can also be involved, and the timing of the assessment should be considered. In particular, infectious epidemic waves can drive peaks of incidence. In Uganda, Kaiser et al. reported a much higher age-adjusted incidence in an onchocerciasis endemic area than in nonendemic areas (232 vs. 77 per 100,000 person-years) [14].

Compared to studies on incidence, those on prevalence are more largely available. These mostly employ cross-sectional designs (e.g., door-to-door surveys with standardized validated screening questionnaires) that are easier and less expensive compared to cohort studies.

Fiest et al. estimated an overall lifetime prevalence of epilepsy of 7.60 per 1000 population (95% CI 6.17–9.38), higher in LAMICs (8.75 per 1000) compared to HICs (5.18 per 1000). Smaller differences have been observed for point prevalence (6.68 vs. 5.49 per 1000) [11]. Studies from LAMICs tend to show a high prevalence in adolescents and young adults, with much lower figures in the elderly [17], resembling the incidence pattern. As mentioned above, the demographic structure of the study population, the prevalence of environmental risk factors, and the frailty of the local health system can be implicated. However, like studies on incidence, prevalence studies in Africa can exhibit significant heterogeneity between countries and even within the same country. Garrez et al. recently reported a very high epilepsy prevalence by a door-to-door survey performed in southern rural Rwanda. They reported a significant lifetime prevalence (76.2 per 1000 individuals) and emphasized the importance of carefully screening for nonconvulsive seizures, which may go unnoticed. Notably, approximately one-fifth of the cases exhibited only nonconvulsive seizures [18]. The diagnostic difficulties for nonconvulsive epilepsy have also been reported in a recent study performed in Kenya [19].

In the systematic review by Preux and Druet-Cabanac, the median prevalence was 15 per 1000 (range 5.2–70.0) in Sub-Saharan Africa [12]. Many of the included studies were small and differed in methodology; focusing on the wider five studies (>15,000 subjects), and prevalence ranged from 5.3 to 12.5 per 1000 [12]. The definition of epilepsy was not systematically provided and only some studies focused on active epilepsy with precise criteria (i.e., regular treatment with antiseizure medications or when the most recent seizure has occurred within the last 5 years) [21].

Recently, Biset et al. performed a meta-analysis on the epidemiology of epilepsy in Africa, focusing on children and adolescents. They found a pooled prevalence of cumulative epilepsy of 17.3 per 1000 children (pooled prevalence of active epilepsy = 6.8; lifetime epilepsy = 18.6 per 1000 children) and a pooled incidence of 2.5 per 1000 children. The highest prevalence of epilepsy was reported in Southern African countries, with a rate of 129.3 per 1000 children, followed by Central African countries, with a rate of 32.3 per 1000 children, and Northern African countries, at 24.1 per 1000 children [20].

### 3.2. Natural History, Prognosis, and Mortality of Epilepsy

The natural history of epilepsy can be partially understood through epidemiological studies conducted in resource-poor settings. Epilepsy is generally considered a treatable condition, with up to 80% of people achieving prolonged periods of seizure remission. In some cases, individuals may even discontinue treatment and remain seizure-free. Based on epidemiological evidence, Sander proposed four distinct courses for PWE: (i) Excellent prognosis (20–30%): High likelihood of spontaneous remission, potentially not requiring treatment. (ii) Good prognosis (30–40%): Seizures are typically well controlled with medication, and treatment may be discontinued after a period of remission. (iii) Uncertain prognosis (10–20%): Seizures may respond to medication, but there is a higher chance of relapse after treatment withdrawal. (iv) Poor prognosis (20%): Seizures are less likely to respond to medication, with a higher risk of recurrence [22].

The disease course varies according to the underlying cause, seizure type, and other less predictable factors. The prognosis of untreated epilepsy can be reliably assessed only in resource-poor settings where epilepsy is often untreated [23]. The prognosis can be inferred by the comparison of the prevalence of active epilepsy to the incidence of epilepsy in untreated PWE. In Ecuador, Placencia et al. estimated a remission rate of at least 50% [24]. Similar findings have been obtained in Africa, in a rural area of Malawi [25]. In spite of the limited access to treatment, remission rates of epilepsy are comparable to those observed in HICs [4]. However, these data need further confirmation due to the circumstantial nature of the evidence, the use of nonstandard case definitions, and the lack of homogeneous investigational procedures. In Africa, the proportion of people with active epilepsy not currently receiving any antiseizure medication (ASM) treatment, or not currently receiving adequate ASM treatment (i.e., “treatment gap”) is very high, with a median value of 80%, and up to 100% in Togo and Uganda [26]. PWE suffer a higher mortality rate than those without epilepsy. Causes of death in PWE can be divided into epilepsy-related (e.g., sudden unexpected death in epilepsy, status epilepticus, drowning) and non-epilepsy-related deaths (e.g., cerebrovascular disorders). Avoidable deaths in PWE are often caused by the epilepsy itself, as the increased risk of death over the general population can be eliminated by achieving seizure freedom through effective treatment strategies. Non-epilepsy-related deaths are, conversely, due to underlying disorders or to comorbidities and cannot be directly modified by addressing the epilepsy course. According to Mbivzo et al., the standardized mortality ratio (SMR) worldwide is higher for epilepsy-related causes (median 3.8) than for unrelated causes (median 1.7), suggesting that more PWE are likely to die prematurely from the first category [27]. In LAMICs, about half of cases are due to epilepsy-related causes, especially status epilepticus and possible or probable sudden unexpected death in epilepsy, followed by accidents [28].

People living in Africa experience the highest SMR (range 2.6–7.2, median 5.4). SMRs are remarkable in children and young adults possibly due to both the comparatively lower mortality rates in the general African populations of young age and to the remarkable risk of death associated with symptomatic or structural/metabolic etiologies. In fact, these etiologies (e.g., traumatic brain injury, possibly arising from vehicular collisions, occupational accidents, sports, violence) are more common in males, who exhibit a higher risk than women. Males may also be more exposed to hazardous occupations, yielding increased risk of drowning, falls, or other fatal injuries consequent to seizures [28]. Drowning appears to be a leading cause of death for PWE in Africa [27]. Mortality due to indirect causes (accidents, drowning, burns) are relatively more common in Africa than in HIC and should be prevented through education and safety measures [28].

### 3.3. Seizure Classification

From 2013 to 2023, there were only ten papers that reported data on seizure classification among children with epilepsy (CWE) in SSA (Table 2). To provide a more comprehensive picture, we included an additional paper by Burton published in 2012 in our review [29]. The studies we assessed were all retrospective and observational, except for a literature review conducted by Samia et al. in 2019 [30], mostly undertaken in Kenya and South Africa in cohorts ranging from 76 [31] to 2407 PWE [32].

A slight male predominance was observed in most studies, except for one that reported a higher proportion of females. Regarding seizure classification, the 2017 International League Against Epilepsy (ILAE) position papers’ suggestions were fully applied in its extended version only by Ackermann et al. and Aricò et al. [32,33]. The remaining papers utilized less detailed classifications, sometimes combining seizure classification with epilepsy and syndrome classification. Published studies exhibited a significant heterogeneity in terms of seizure semiology, with varying percentages reported for different seizure types. Focal seizures ranged from 8.2% to 77.7% in the descriptive articles, and from 13.1% to 78.6% in the review article by Samia et al. Generalized motor seizures were often poorly defined, but generalized tonic–clonic (GTC) seizures were commonly acknowledged across multiple studies [30,31,32,33,34,35]. Myoclonic, tonic, atonic, and clonic seizures were reported in a limited number of papers [29,31,32,33,34], and epileptic spasms were mentioned in three studies [32,33,36]. Generalized nonmotor seizures (absence seizures) were cited in five papers [29,32,33,34,37], while focal nonmotor seizures were mentioned in only two studies [32,33]. Awareness during focal seizures was considered in five articles [29,33,34,35]. Regarding the classification of epilepsy, in Sub-Saharan Africa, most epilepsies have been classified as focal (50–65%), resembling the figures observed in HIC (60–70%). The definition of the type of epilepsy rather than seizures is, however, more difficult and often requires a longitudinal observation [4,12,22].

Most of the reviewed papers presented electroencephalogram (EEG) findings, with varying rates of EEG abnormalities reported (20–89%). However, the classification of these abnormalities differed remarkably, making it challenging to compare results. Few studies included neuroimaging data, primarily consisting of CT scans, which provided additional insights into the neuroradiological correlates of epilepsy.

**Table 2:** Overview of articles reporting data on semeiology of epileptic seizure and classification. The table summarizes the data on clinical manifestations of seizures, availability of diagnostic procedures (by EEG and CT or MRI scan) in different Sub-Saharan African countries. CWE = children with epilepsy, SeLECTS = Self-limited epilepsy with centrotemporal spikes, DALYs = disability-adjusted life, PWE = people with epilepsy, CSE = convulsive status epilepticus, ILAE = International League Against Epilepsy, MRI = magnetic resonance imaging, EEG = electroencephalogram, CT = computerized tomography.

Scheme	Primary Focus and Population	Seizure/Epilepsy Classification	EEG	Neuroimaging	Limitations
Burton et al., 2012 [29]	Tanzania. Prevalence and risk factors for epilepsy in children: 112 CWE age 6–14 y (males 50.9%).	- Focal motor onset with secondary generalization 73 (65.2%). - Generalized motor onset tonic–clonic and clonic 19 (16.9%). - Focal onset with impaired awareness 11 (9.8%). - Generalized motor myoclonic 2 (1.8%) - Focal motor 3 (2.7%). - Generalized non motor onset (typical) 1 (0.9%). - Unclassified 3 (2.7%).	Performed in 101 patients. - EEG anomalies in 44 patients (43.6%), particularly: - Generalized epileptiform abnormalities 9 (20.5%). - Multifocal epileptiform abnormalities 11 (25%). - Temporal lobe abnormalities 7 (15.9%). - Extratemporal focal abnormalities 9 (20.5%). - Generalized non-epileptiform abnormalities 8 (18.1%).	CT performed in 90 patients (80.3%). Neuroradiological anomalies in 26 patients (28.9%), particularly: - Focal cerebral atrophy 5 (19.3%). - Cerebellar/ brainstem atrophy 4 (15.4%). - Porencephalic cyst 2 (7.7%). - Generalized lack of white matter bulk 3 (11.5%). - Calcified lesion 2 (7.7%). - Neurocysticercosis 2 (7.7%). - Pre/perinatal vascular event 5 (19.3%). - Previous tuberculous meningitis 1 (3.8%). - Sturge–Weber 1 (3.8%). - Tuberous sclerosis 1 (3.8%).	- Small sample. - No data for age groups. - Seizure classification rough.
Lagunju et al., 2015 [37]	Nigeria. EEG impact on epilepsy care in children referred to a pediatric neurology clinic with suspicion on epilepsy. CWE 329 age: 3 mos–16 y: males = 59.9%.	- Generalized onset 151 (48.7%). - Focal onset 57 (18.4%). - Focal onset with secondary generalization 47 (15.5%). - Generalized nonmotor onset (typical) 25 (8.1%). - Infantile epileptic spasm syndrome (IESS) 16 (5.2%). - SeLECTS 10 (3.2%). - Lennox–Gastaut syndrome 2 (0.6%). - Dravet syndrome 2 (0.6%).	Performed in 329 patients. EEG anomalies in 108 (32.82%), particularly - Continuous generalized slow wave activity 75 (69.4%). - Diffuse slow wave activity with a poorly organized background 14 (13%). - Asymmetry 11 (10.2%). - Intermittent regional slow wave activity 6 (5.6%). - Beta waves 2 (1.8%).	Not performed.	- Epileptic syndromes included into epilepsy classification. - No data for age groups. - EEG findings imprecise. - Seizure classification rough.
Kariuki et al., 2024 [38]	Kenya. Determine incidence, DALYs, risk factors and causes of admissions in PWE in a rural hospital. 743 CWE age: 0–13 y, males = 60%.	- Generalized onset seizures 646 (86.94%). - Focal onset seizures 97 (13.1%). - “Complex seizures” (focal onset, prolonged and/or repetitive) 427 (58%).	Not performed.	Not performed.	- No diagnostic tools used. - No data for age groups. - Seizure classification rough.
Reddy et al., 2017 [31]	South Africa. Pediatric CSE. 76 CWE age: 1 mo–13 y, males 62%.	- Generalized motor tonic–clonic 40 (53%). - Focal motor onset seizures evolving in CSE 14 (18%). - Generalized motor tonic 3 (4%) - Unknown onset 19 (25%).	Performed in 44 (58%) patients. Findings: - EEG anomalies in 39 (89%) Particularly: - Epileptiform activity 4 (10%) - Low amplitude pattern 4(10%) - Electro-cerebral silence 4(10%) - Burst suppression 3 (8%) - Diffuse beta activity 1 (3%)	Performed in 70 (92%) patients: - MRI: 25 (36%) patients. - CT: 68 (97%) patients. Findings: - Neuroradiological anomalies 53 (76%), particularly - Hypoxia. - Hypoperfusion-related changes 17 (32%). - Arterial infarct 7 (13%). - Leptomeningeal enhancement 6 (11%). - Cerebral oedema 6 (11%). - Cerebral atrophy 5 (9%). - Venous thrombosis/venous infarcts 4 (8%). - Other abnormalities 8 (16%).	- Small sample. - Incomplete documentation for some records. - No data for age groups. - Seizure classification rough.
Matonda-ma-Nzuzi et al., 2018 [36]	Congo. Behavioural problems and cognitive impairment in CWE attending a mental health center. 104 CWE age: 6–17 y, males 58.6%.	- Focal onset seizures 58 (55.8%). - Generalized onset seizures 35 (33.6%). - Generalized motor epileptic spasms 3 (2.9%). - Unclassified 8 (7.7%).	Not performed.	Not performed.	- Small sample. - No diagnostic tools used. - No data for age groups. - Seizure classification rough.
Lompo et al., 2018 [39]	Burkina Faso. Etiology of nongenetic epilepsies in CWE. 115 CWE age 0–18 y, males 62.6%.	- Focal onset epilepsy 70 (60.9%). - Frontal onset 33 (28.7%). - Central onset 15 (13%). - Temporal onset 14 (12.2%). - Occipital onset 8 (6.9%). - Generalized onset epilepsy 22 (19.1%). - Focal onset to bilateral tonic-clonic 13 (11.3%). - Undetermined 10 (8.7%).	Epileptic paroxysmal anomalies 86 (74%)	Performed: - MRI: 11 (9.6%) patients. - CT: 104 (90.4%) patients. Findings: Neuroradiological anomalies 72 (62.2%), particularly - Cortico-subcortical atrophy 55 (47.8%). - Circumscribed hypodensity 18 (15.6%). - Cortico-subcortical calcifications 5 (4.3%). - Porencephalic cavity 12 (10.3%). - Chronic hydrocephalus 4 (3.5%). - Heterogeneous nodules under ependymal 2 (1.7%). - Hippocampal sclerosis 3 (2.6%). - Cortical development malformations 3 (2.6%). - Brain tumor 2 (1.7%).	- Small sample. - No data for age groups. - EEG findings imprecise. - Seizure classification rough.
Ahmad et al., 2018 [34]	Nigeria. Clinical seizure types and EEG findings in CWE seen in a pediatric neurology clinic. 303 CWE age: 3 mos–15 y, males 65.5%.	- Generalized motor onset tonic–clonic - 192 (63.4%). - Generalized motor onset myoclonic 31 (11.9%). - Generalized motor mixed forms 31 (10.2%). - Focal onset to bilateral tonic-clonic 18 (5.9%). - Generalized motor onset atonic 9 (3%). - Focal motor onset 7 (2.3%). - Generalized non motor onset (typical) 3 (1%). - Epileptic syndromes 7(2.3%).	Performed in 176 (58.1%) patients. Epileptiform discharges found in 146 (83%) records.	Not performed.	- Old classification used. - Epileptic syndromes included into seizure classification. - No data for age groups. - Seizure classification rough. - EEG findings imprecise.
Ackermann et al., 2019 [32]	South Africa. Epidemiology of CWE referred to a tertiary service. 2407 CWE age: 0–12, males 56%.	Seizure types grouped by age and more detailed classification. - Generalized onset 1056 (52%). - Focal onset 1309 (54%). - Unknown onset 244 (10%).	Not performed.	Not performed.	- No data from diagnostic tool.
Egesa et al., 2022 [35]	Kenya. To Review seizure semiology and etiological data to fit ILAE-2017 criteria. 256 CWE, males 43.2%.	Seizure type: - Focal onset 299 (61.9%). - Generalized onset 157 (32.5%). - Unknown onset 27 (2.6%). - Seizure subtypes: - Generalized motor onset tonic–clonic 31 (44.9%). - Focal aware onset 42 (46.7%). - Focal impaired aware onset 57 (62%). - Focal to bilateral tonic-clonic 42 (47.2%).	Performed. EEG anomalies 122 (60.1%).	Performed in 22% of the whole sample. No data on results.	- Retrospective analysis. - No data for age groups. - Seizure classification rough. - EEG findings imprecise.
Samia et al., 2022 [9]	Childhood epilepsy care in Kenya and knowledge gap.	- Generalized motor onset tonic–clonic 33.6–70.4%. - Focal onset 13.1%–78.6%.	Any abnormality 20–39%. - Focal abnormalities up to 61.6% of abnormal EEGs. - 3 Hz spike-wave 7.4% of abnormal EEGs. - Hypsarrhythmia 2.8% of abnormal EEGs.	One study of neuroimaging included. No abnormal findings on 11 children with focal epilepsy and loss of awareness.	- No data for age groups. - Seizure classification rough. - EEG findings imprecise.
Aricò et al., 2023 [33]	Impact of a newly established clinic for pediatric epilepsy. 143 CWE age 0–18; males 57.3%.	Seizure type: - Generalized motor onset tonic 87%. - Generalized motor onset clonic = 82%. - Generalized motor onset tonic-clonic 29%. - Focal motor onset automatism 14%. - Generalized motor onset myoclonic 10%. - Generalized non motor onset (cognitive, atonic, absences, behavioural arrest, epileptic spasms, sensory): reported in less than 10% of cases. Epilepsy classification: - Focal with motor onset and impaired awareness 51%. - Motor generalized 24%. - Focal with motor onset and preserved awareness 8%. - Focal with nonmotor onset and impaired awareness 6%. - Motor with unknown onset 5%. - Non motor generalized (CAE) 3%.	EEG performed on 48 cases. Findings: - Normal EEG 9 (18.7%) records. - Background abnormalities 27 records. - Interictal abnormalities 34 records. - Ictal abnormalities 8 records (5 focal, 3 diffuse). - Focal abnormalities (ictal or interictal) 19 (39.6%) records. - Multifocal abnormalities (ictal or interictal) 9 (18.7%) records. - Diffuse abnormalities (ictal or interictal) 7 (14.58%) records. - EEG led to diagnosis changes in 29.2% of patients and to treatment switch in 31.2% of patients.	Not performed.	- Retrospective analysis. - Small sample. - No data for age groups.

### 3.4. Etiology

Epilepsy is a symptom of an underlying neurologic disease; indeed, the etiology of epilepsy is a major determinant of clinical course and prognosis [40]. The new classification of epilepsies from ILAE 2017 incorporates etiology along each stage of diagnosis, as it often carries significant treatment implications. The new classification recognizes six etiological subgroups, selected because of their potential therapeutic consequences: structural, genetic, infectious, metabolic, immune, and unknown groups [41].

The etiological profile of epilepsy in SSA significantly diverges from HIC. There is a higher prevalence of structural causes, possibly reflecting a greater burden from trauma and brain infections, as well as a marked increase in infectious causes influenced by endemic diseases.

Determining the causes of epilepsy typically demands the use of various diagnostic tools like magnetic resonance imaging (MRI), EEG, genetic tests, and metabolic assessments. Unfortunately, these resources are often scarce in Sub-Saharan Africa, limiting the chance of obtaining conclusive data. Parasitic diseases (malaria cysticercosis, onchocerciasis, toxocariasis, and toxoplasmosis), perinatal events, head injuries, HIV infection, and hereditary factors can all be relevant provocative agents [42]. In 2014, Ba-Diop et al. published a comprehensive review on Lancet neurology on epidemiology, causes, and treatment of epilepsy in SSA, identifying family history of seizures, previous febrile seizures, perinatal trauma, head injury, and central nervous system (CNS) infections as main risk factors [43]. After that, some national-based studies in SSA were published. We found 11 particularly relevant studies (Table 3). Overall, structural or infectious etiologies appear to play a major role in Africa [30,35,39,42,44]. Specifically, structural causes readily include the sequelae of adverse perinatal events or cranial trauma [39], while infectious causes encompass a former history of meningitis, cerebral malaria, or exposure to specific parasites [30,45,46]. A growing body of epidemiological research indicates that onchocerciasis may induce seizures through direct or indirect mechanisms, giving rise to the concept of onchocerciasis-associated epilepsy (i.e., nodding syndrome, see below) in affected areas [47,48]. Of note, this has been highlighted as a significant but overlooked public health crisis, especially in Sub-Saharan Africa, during the 2nd International Workshop on onchocerciasis-associated epilepsies (OAEs) in Antwerp, Belgium, September 2023 [49]. A large amount of data suggest a strong association between onchocerciasis and epilepsy—especially regarding O. volvulus and higher microfilarial loads. Treating onchocerciasis by ivermectin also reduces the seizure burden [50,51,52,53]. A recent brain MRI study performed in Kenyans and South Africans with active convulsive epilepsy revealed structural abnormalities in 59–65%, mostly attributable to hippocampal sclerosis and gliosis [38]. Apolot et al. had formerly described similar figures in Uganda, also disclosing a correlation between the presence of abnormal EEG findings and diagnosis of morphological brain abnormalities [54].

However, these findings may not fully represent the actual spectrum of epilepsy-inducing conditions in Africa, but, rather, the diagnostic opportunities available. In the past decade, no data on genetic or metabolic epilepsies in SSA were published until 2022. Since then, two prospective studies employing molecular testing on a cohort of patients with developmental and epileptic encephalopathies (DEE) have been performed in South Africa. Essajee et al. revealed a noteworthy diagnostic yield of next-generation sequencing (NGS) analysis, identifying pathogenic variants in 18/41 (43.9%) patients by a panel of 308 genes [56]. Esterhuizen et al. analyzed a cohort of children with DEE by means not only of a gene panel built on 71 DEE-associated genes, but also by chromosomal microarray and exome sequencing. They found pathogenic variants in 41/234 subjects (19%), especially in those with neonatal/early infantile onset, neuropsychiatric comorbidities, and somatic dysmorphisms. In these high-risk subjects, the authors opted for genetic investigations based on a “Think-Genetics” decision tree, which is particularly suited to low-income settings. The first level of analysis involved small gene panels or chromosomal microarrays, with the latter reserved for those with a history of intellectual disability or dysmorphisms. Only unresolved cases were subjected to exome sequencing, a technique accessible to only a fraction of patients in Sub-Saharan Africa. Of the 41 children with pathogenic/likely pathogenic findings, 26 had variants with treatment implications [57]. Those studies revealed a noteworthy diagnostic yield of genetics, suggesting that genetic epilepsies are consistently underdiagnosed in SSA.

### 3.5. Nodding Syndrome

In recent years, there has been growing interest in an acquired neurological disorder called nodding syndrome (NS), characterized primarily by severe attacks of nodding of the head, followed by behavior difficulties and psychiatric disorders, declining cognitive function, stunting and growth failure, delayed puberty, and physical and motor disability [58,59]. NS commonly appears in children aged 5 to 15 years old [60], often following prodromal symptoms observed two years prior to the onset of head nodding. These early signs include periods of blank staring, inattentiveness, complaints of dizziness, lethargy, and general weakness [58]. As Abd-Elfarag et al. point out in their systematic review, nodding syndrome is a severe neurological disease, and while its precise cause is still under investigation, studies have explored various factors such as infections, autoimmune responses, and nutritional deficiencies [61].

During a nodding episode, a sudden loss of muscle tone occurs, causing the head to drop forward toward the chest. This happens at a rate of about 5–10 drops per minute. Ictal EEG shows generalized electrodecrement, and paraspinal electromyography indicates a dropout consistent with an atonic seizure [62]. The head nod represents the main and most characteristic symptom of the condition. However, patients frequently experience concurrent myoclonic jerks and atypical absences and may develop additional seizure types over time, including tonic–clonic and myoclonic seizures [58]. The condition usually progresses with cognitive decline, behavioral disturbances, especially learning and memory difficulties, temper tantrums, and depression. Some patients may develop limb deformities and spine abnormalities [58]. Brain MRI studies showed varying degrees of cortical and cerebellar atrophy, along with frontal subcortical gliosis in some patients as well as hippocampal atrophy and sclerosis [60].

NS treatment includes antiseizure medications, nutritional support, and psychosocial interventions to manage symptoms and improve quality of life. Epilepsy prognosis is quite poor. Seizures are usually managed with common ASMs, such as sodium valproate, carbamazepine, phenytoin, and phenobarbital, leading to seizure freedom in 0–25% of cases [60,63]. A recent cross-sectional observational study has indicated that phenytoin may be more effective in controlling head nodding compared to monotherapy with other ASMs. However, these findings necessitate further validation through additional research [64].

The cause of NS is still unknown, but several factors have been proposed both as causative or predisposing: (i) Infections: There is a strong relationship between nodding syndrome and infection with the parasite Onchocerca volvulus. This is supported by several observations: no cases of nodding syndrome have been found in areas not endemic for O. volvulus [60]. A higher rate of infection with O. volvulus was found in PWE compared to nonepileptic controls, O. volvulus was identified as a predictor of epilepsy later in life, and successful onchocerciasis elimination strategies have reduced the incidence of epilepsy in regions endemic for onchocerciasis [65]. Despite that, the microfilaria in the CNS are exceedingly rare and there is no documented incidence of O. volvulus in brain tissue [60]. (ii) Autoimmune response: It has been proposed that O. volvulus infection could trigger neuroinflammation and neurotoxicity by an immune-mediated response. Particularly, a strong cross-reactivity was found between Leiomodin-1 antibodies (an action-binding protein increased in patients with NS) and O. volvulus antigens, creating a cycle of injury and neuroinflammation [60]. (iii) Malnutrition: Vitamin B6 deficiency was found to be a main risk factor for NS [66]. In families with one or more NS cases, a history of shortage and consumption of moldy maize was notably more prevalent before the onset of head nodding [67]. (iv) Genetics: An association was found between NS and both protective HLA haplotype (HLA-B*42:01, C*17:01, DRB1*03:02, DQB1*04:02 and DQA1*04:01), and susceptible motif (Ala24, Glu63 and Phe67), in the HLA-B peptide-binding groove. Those findings suggest that immunogenetic fingerprints in HLA peptide-binding grooves tentatively associate with protection or susceptibility to NS [59]. (v) Neurodegenerative condition: There is speculation that NS could represent a form of tauopathy since postmortem studies revealed filamentous tau-positive deposits in the neocortex, in the locus coeruleus, in the substantia nigra, and tegmental nuclei, and since neurological decline is observed in most patients [68].

NS represents an enigmatic and intricate condition characterized by the onset of epilepsy in childhood and a progressive neurological decline. Its etiology involves a multifaceted interplay of factors. Notably, NS presents distinctive traits aligning it with DEE, a heterogeneous group of conditions characterized by early-onset severe epilepsy, EEG abnormalities, and developmental impairment that tends to worsen as a consequence of epilepsy [41]. Indeed, as observed by Mazumder et al., NS shares EEG features with late-onset spasms and Lennox–Gastaut syndrome such as ictal slow waves followed by electrodecrement responses and ictal cortical gamma rhythms, suggesting involvement of widespread epileptic networks across cortical and subcortical structures [53]. Given the age of onset, the progressive clinical trajectory, and distinctive electroencephalographic features, it is plausible to categorize NS as a novel variant of DEE, warranting a multidisciplinary approach to its management. On the other side, a recent autoptic study performed in Uganda has revealed tau pathology in NS, with a peculiar pattern of superficial cortical accumulation, largely involving gyral crowns, that allows distinction from the other tauopathies. The cause of the tau pathology in NS has not yet been established [68].

### 3.6. HIV-Related Epilepsy

Children under 15 years of age with HIV infection are estimated to be 2.6 million worldwide, and the greater part lives in LAMICs (primarily Sub-Saharan Africa and Southeast Asia) [69]. NeuroAIDS is a complication of HIV infection, and the term refers to the spectrum of central and peripheral nervous system effects caused by the virus. Neurological complications in children can be either a direct consequence of HIV infection (such as neuronal damage, cell death) or of secondary conditions (opportunistic infections, cerebrovascular disease, malignancies) [69], or they can result from the indirect effects of HIV including social stressors, poverty, illness, and trauma [70]. Epilepsy and seizure are among the most frequent CNS complications of HIV infection. At the moment, the precise prevalence of epilepsy in children with HIV infection in Africa is not clear; according to [69], it is 7.6–14%. It is possible that many regional differences, partially related to the risk of becoming infected, the availability of antiretroviral therapy (ART) and ASMs, make the evaluation difficult. From 2013 to 2023, there were six papers regarding epilepsy in patients with HIV infection/AIDS in Africa (five original research and a chapter taken from the Handbook of Clinical Neurology). All the other original research was either longitudinal or retrospective observational studies, including from 29 to 2137 subjects according to the inclusion criteria (e.g., HIV-infected children, HIV-infected children with new-onset seizure) (Table 4). All the data derive from SSA countries (Zambia, South Africa, Uganda, Botswana). Most studies had a slight male predominance. Different aspects of epilepsy in HIV-infected children have been explored, ranging from the clinical profiles of HIV disease in children to the prevalence of epilepsy (and other neurological disorders) in HIV-infected children and correlation with being on ART.

According to the semiology, there was a predominance of generalized tonic–clonic seizures (51–67.3%); status epilepticus was commonly reported (21.2% according to Burman et al. [71], 37% according to Ravishankar et al. [72]). The most common etiologies for epilepsy were CNS infections (opportunistic and nonopportunistic) followed by HIV neurotoxicity, structural lesions, metabolic disturbances, and birth asphyxia. According to the WHO stage of HIV disease, 60–76% of CWE were in an advanced stage of the disease (WHO stage 3 or 4), with the exception of one study [70], in which the majority of participants (55.7%) were on WHO stage 2. There was great variability in the diagnostic evaluation. Neuroimaging (CT scan and/or MRI) was more likely performed in children with HIV disease and epilepsy compared to children with no epilepsy, but these opportunities were mainly available in urban hospitals where there was the possibility of obtaining an EEG recording. Antiepileptic treatment was investigated in only two papers [71,73]. Both assessed that sodium valproate was the most frequently ASM used. Seizure control was investigated in only one paper and it emerged that 24 out of 49 patients became seizure-free while 8 out of 49 patients had significant reduction in seizure frequency. Burman et al. investigated the possible interactions between ASMs and ART (specifically Efavirenz) that may have influenced the seizure control. It ended up that children with poor control of their seizures were more likely treated with Efavirenz, which is an inhibitor and inducer of CYP3A4, a crucial enzyme in the ASMs metabolism [71]. Surprisingly, former studies had shown that EFV has no effect on the metabolism of ASMs. Further investigations regarding responsibility of drug interactions on seizure control are needed.

In conclusion, all the reviewed papers agreed that epilepsy is a common neurologic complication of HIV infection, that more likely affects [74] children in advanced stage of disease and that can worsen the prognosis. In one reviewed paper [73], 75% of the children enrolled were diagnosed with epilepsy before or when starting the ART; this suggests that the presence of clinical manifestations of epilepsy could have caused further investigations and accelerated the disclosure of an HIV-infection. Bearden et al. assessed that being on early treatment with combined antiretroviral therapy (cART) can reduce the odds of epilepsy by reducing the rates of CNS infections and HIV neurotoxicity [75]. Therefore, although the optimal timing for starting cART in LAMICs is still unknown, early treatment appears to be protective against neurological complications and, indirectly, against epilepsy itself.

**Table 4:** Associations of HIV infection and seizures. Overview of the articles reporting data on epilepsy in children with HIV infections comparing the epidemiology, etiology, and characteristics of the used medications. HIV = human immunodeficiency virus, CT = computed tomography, MRI = magnetic resonance imaging, EEG = electroencephalogram, CWE = children with epilepsy, CNS = central nervous system, WHO = World Health Organization, ART = antiretroviral therapy, CSF = cerebrospinal fluid, ASMs = antiseizures medications, VPA = valproic acid, LTG = lamotrigine, LEV = levetiracetam, LCM = lacosamide, GBP = gabapentin, PGB = pregabalin, GTC = generalized tonic–clonic, TB = tuberculosis.

Study	Population	Study Design	Seizure/Epilepsy Classification	Diagnostic Evaluation	Etiology	HIV WHO Stage	ART and/or ASMs	Main Findings
Bearden et al., 2015 [76]	Botswana; 29 HIV CWE aged 0–18 years and 58 matched controls.	Observational retrospective, case–control.	Not mentioned.	- Cranial CT: 20. - Brain MRI: 4. - CSF: 15. - EEG not available.	- CNS infections (tuberculosis meningitis, cryptococcal meningitis, bacterial meningitis, toxoplasmosis) 31%. - HIV neurotoxicity 27%. - Unknown 31%. - Other (ischemic stroke, congenital malformation, birth asphyxia) 9%.	At enrollment: Cases WHO stage 4 66%. Controls WHO stage 4 38%.	All participants on cART during the study (median age 70 months for both case and controls). 39 patients (45%) received early treatment (8 cases and 31 controls). Not mentioned ASMs.	Early treatment with cART is likely to be protective against epilepsy in children with HIV.
Wilmshurst J.M. et al., 2018; Chapter 8 of the Handbook of Neurology [71]	Children <15 years in Sub-Saharan Africa.	Review.	Focal onset seizures are more likely than generalized ones.	- Clinical assessment. - Laboratory investigations. - Chest X-ray. - Cranial CT—CSF.	May be related to HIV damage or secondary to acquired pathology (neuroinfection).	Not mentioned.	- ASMs is often limited. - Drug–drug interactions with cART. - VPA, LTG, LEV, LCM, GBP, PBG are the favored ASMs. VPA and LTG are the first-line choice, LEV is an alternative.	Main features of epilepsy in HIV patients.
Mpango et al., 2019 [72]	Uganda; 1070 participants: 677 (63.3%) aged 5–11 years and 393 (36.7%) adolescents aged 12–17 years (48% males). 43 (4%) with probable epilepsy.	Observational longitudinal.	Not mentioned.	Not mentioned.	Not mentioned.	596 participants (55.7%): stage 2.	1024 (95.7%): on ART. Not mentioned ASMs.	Prevalence of neurological disorders (enuresis/encopresis, motor or vocal tics, epilepsy) among children with HIV was 18.5%. Prevalence of probable epilepsy was 4%.
Burman et al., 2019 [73]	South Africa; 227 participants (10–176 months), 131 males (57.7%); 23% at least one seizure, 14.2% diagnosis of epilepsy.	Observational retrospective, case–control.	- Generalized seizures 35 CWE (67.3%; most frequent: GTC). - Status epilepticus 11 CWE (21.2%). - Focal seizures 17 CWE (32.7%).	- EEG 63.5% had 56%. - Brain scan: 100% in the seizure’s group more likely to have CT (69.4%) than MRI (44.6%).	- CNS infections 61.5%. - Unknown 28.8%. - Structural 9.6%.	69%: stage 4.	- 98% on ART (median age ART starting: 18 months). - 53% were treated with VPA.	- Data on the epidemiology and complexity of management of seizures in HIV-infected children. - No correlation between stage disease and seizure occurrence. - No correlation between being on ART and age when ART was started with seizure occurrence. - Interactions between efavirenz and ASMs are unlikely to be responsible for poor seizure control).
Michaelis et al., 2020 [75]	South Africa (Eastern Cape) 2137 HIV children (1 month–12 years). 53 (2.5%) with epilepsy; 26 (53%) males.	Observational retrospective study.	Not mentioned.	Not mentioned.	Most common: prior CNS infection (tuberculosis meningitis).	- 76% of CWE: WHO stage 3 or 4. - 80% of children without epilepsy: WHO stage 1.	- 100% started ART during the study. - 75% diagnosed with epilepsy before ART. - 48 out of 49 CWE were treated with VPA (24/49 became seizure free, 8/49 significant reduction in seizure frequency).	- Epilepsy prevalence in children with HIV on ART in a semirural area was 2.5%. - Prior CNS infections and/or HIV encephalopathy and advanced disease were associated.
Ravishankar et al., 2022 [74]	Zambia; 73 children (2.2–10 years) with HIV infection and new-onset seizure; 39 males (53%).	Observational longitudinal.	- Focal seizures 36 (49%). - Multiple recurrent seizures 28 (38%). - Status epilepticus 27 (37%).	- Neuroimaging (39 children). - EEG (12 children).	- Infectious 54% (opportunistic and nonopportunistic infections). - Metabolic 19% (renal failure, hypoglycemia). - Structural lesions 10%.	44 (60%): WHO stage 4.	36 (49%) were on ART regimen (34 on cART); of these, 19 children (56%) on treatment for >1 year. Not mentioned ASMs.	- Despite widespread HIV testing and cART in Zambia, this is not sufficient. - New-onset seizure in children with HIV occur in advanced, active HIV disease. - 22 children (30%) died due to advanced HIV, meningitis, sepsis, disseminated TB, status epilepticus.

### 3.7. Diagnostic Tools

Regarding the diagnostic tools developed and validated in the African population for the investigation of epilepsy, in the selected 10 years’ time frame, five articles were reviewed in this article: on the development and validation of diagnostic questionnaires (n = 3, Table 5 and Supplementary Material), on the impact of screening tools in low-resource countries (n = 1), and on the access to electrophysiology services (n = 1).

The limited availability of laboratory, electroencephalography, and neuroimaging testing mandates us to especially rely on history taking and surveys. Electroencephalography (EEG) is available in various parts of Sub-Saharan Africa, but its accessibility and availability can vary significantly between urban and rural areas. The cost of EEG testing can be a barrier for many patients, Regular maintenance and technical support for EEG machines can be a challenge, affecting their operational availability. Similarly, CT and MRI facilities are more commonly found in major urban hospitals and medical centers, particularly in capital cities, often with prohibitive costs. Functional MRI is even more difficult to obtain, limiting the possibility to develop efficacious programs for epilepsy surgery [77].

Specific diagnostic questionnaires can be extremely useful, especially in rural areas. In their study, Patel et al. proposed a diagnostic questionnaire validated on the pediatric population (6 months to 18 years), in comparison with the other authors that validated the questionnaire from 6 years of age. The tool comprises 18 total questions. The initial questions, in conjunction with three subsequent questions, inquire about the occurrence of potential epileptic episodes. In the event of a positive response to the initial four questions, a diagnosis of epilepsy can be made, thereby indicating that the investigator can proceed with the subsequent 14 questions. The objective of the questionnaire is both to identify CWE (question 1) and to classify the type of seizure into either focal/multifocal seizure (questions 2–13) or generalized seizure (questions 14–15) [76]. Vergonjeanne et al. described two decades of experience with the questionnaire for Investigation of Epilepsy in Tropical Countries (IENT) [78]. The IENT questionnaire includes nine sections ranging from anamnestic data to clinical examination, including a section on etiological investigations and treatment. The first two sections, “demographic data” and “screening”, can be filled by a nonmedical investigator. Jones et al. developed a tool for the diagnosis of convulsive seizures based on binary information administered through a smartphone application. The tool is designed to direct the individual to the most appropriate clinician. A further objective is to assist healthcare professionals in monitoring patients via the application. The screening questionnaire is composed of binary questions (yes or no) and the responses provide the probability and classification (likely or unlikely) of convulsive epilepsy. Upon diagnosis, a clinical report is generated, comprising anonymized metadata and questionnaire responses [79]. All three studies used a two-step approach with a first step characterized by screening participants randomly selected or identified via door-to-door visits with a few screening questions and the administration of a questionnaire by nonmedical healthcare workers. In step two, all individuals screened positive from the questionnaire were invited to be examined by a neurologist. According to Shalu et al., prevalence estimates obtained from the two-stage approach may be seriously biased due to the failure to consider the imperfect validity of the tests, used in the first or second stage, or in both stages, leading to verification bias and misclassification errors. To overcome biases, it is important to compare the collected data with statistically adjusted ones, obtained according to statistical analysis such as the Bayesian latent-class models [55].

**Table 5:** Diagnostic questionnaires. Of the 5 articles reviewed regarding the diagnostic tools, the table shows an overview of the three describing the development and validation of diagnostic questionnaires. SSA = Sub-Saharan Africa.

Study	Population	Type of Study	Intervention	Indicator of Accuracy	Outcome	Strengths	Limitations
Patel et al., 2016 [78]	Tanzania and Zambia; 6 months to 18 years.	Observational longitudinal.	Administered to patient’s caregiver by a nonmedical staff member. 15 most discriminating features of semiology characteristics.	Sensitivity of 78% and positive predictive value of 81.5%.	Discriminate focal from generalized seizures.	Translated into local dialects.	- Difficulties with precise classification of specific seizure semiologies. - Does not include all types of epilepsy. - Does not include questions on concomitant illness and etiologies.
Vergonjeanne et al., 2021 [79]	African population; all ages.	Review and observational longitudinal.	Questionnaire for investigation of epilepsy in tropical countries (IENT). 9 sections with a total of 213 items combining both binary and open answer. The first two sections can be filled by a nonmedical investigator.	The sensitivity and specificity were estimated to be 95.1% (95% CI: 87.3–98.4%) and 65.6% (95% CI: 57.5–72.9%), respectively.	- Estimate prevalence in an area. - Identify clinical forms of epilepsy determine etiologies. - Describe treatment.	Available in several languages (French, English, Spanish, and Portuguese).	- Validated in 2000, need an update according to the new classification of epilepsy. - A semiological support could be added to im-prove the classification of seizures and epilepsy.
Jones et al., 2023 [55]	SSA; population over 6 years.	Observational retrospective, case–control.	Questionnaire for community-based healthcare workers. 8 binary questions.	Sensitivity, specificity, and positive and negative predictive values were 97.5% (93.7–99.3), 82.4% (71.2–90.5), 92.9% (87.9–96.3), and 93.3% (83.8–98.2; Table 2).	Diagnosis of convulsive epilepsy.	- Free app Epilepsy Diagnostic Companion (EDC). - Facilitating more appropriate onward referral to a neurologist.	- Convulsive seizures only. - Regional limits. - Difficulties in follow-up participation. - Selection bias. - Prevalence bias.

In their last study, Kander et al. investigated the access and the level of competence of the practitioner of electrophysiology services in SSA, with a special focus on pediatric electrophysiology. As gold standard, EEG study performed by a neurophysiology technologist and interpreted by a specialist with formal training in epileptology can assist and enhance the diagnosis, delineation of syndromes, and, therefore, the management of epilepsy. According to the study, in terms of pediatric EEGs, the survey could not address evidence for improved care; nevertheless, most participants agreed that a viable training model would be beneficial to improve diagnosis and management for pediatric patients [80]. 

Interestingly, the EPInA Study Group has recently developed and validated the Epilepsy Diagnostic Companion, a predictive modeling app designed to confirm convulsive epileptic seizures in individuals with suspected epilepsy. Tailored to specific cultural and regional contexts, it offers a simple questionnaire that can be administered by nonspecialist healthcare workers. This tool has the potential to significantly enhance early diagnosis and subsequent care for PWE through iterative updates [79].

## 4. Future Challenges and Study Limitations

Sub-Saharan Africa faces a significant shortage of healthcare professionals, especially pediatric neurologists. Many SSA countries have fewer than 0.03 neurologists per 100,000 people, compared to 4–8 per 100,000 in most developed countries. The shortage is particularly severe in rural areas, where access to any form of specialized care is limited. A 2017 report indicated that some countries in Sub-Saharan Africa, such as Malawi, have fewer than five neurologists serving the entire country, while other countries like Liberia and Sierra Leone have no practicing neurologists at all. Only a small percentage of these neurologists specialize in pediatric neurology. The low ratio of neurologists is compounded by a lack of other specialized healthcare workers such as pediatricians, nurses, and general practitioners trained to manage epilepsy [77]. A partial support may be obtained by telemedicine, especially for the interpretation of diagnostic exams performed locally and interpreted by expertise outside the continent [81], and promising data exist for low-cost portable EEGs [82]. Active epilepsy may also be difficult to treat for the treatment gap. The only widely available antiseizure medication in most nations is phenobarbital, phenytoin, carbamazepine, and valproate, which are substantially more expensive. Moreover, patients often struggle with adhering to treatment regimens because they may not fully understand the need to take daily medication for a condition that only causes symptoms intermittently [81]. We should also consider that the limitation in diagnostic resource may lead to misdiagnosis of functional/dissociative seizures (formerly known as psychogenic nonepileptic seizures) as epilepsy, and often both conditions may be attributed to supernatural causes, such as witchcraft or spiritual possession leading patients to seek help from traditional or religious healers rather than medical professionals [83,84].

Therefore, providing most medical resources for clinical assistance and scientific research is a major effort, also considering that different regions have to face specific difficulties.

In comparison to LMICs in Asia and Northern Africa, people from SSA have to face higher prevalence rates, and children have major difficulties in accessing healthcare infrastructure and diagnostic tools [84,85].

The heterogeneity of available studies prompted us to perform a scoping review, limited to diagnosis and classification. We attempted to summarize systematic and anecdotal data available in the literature. However, many issues remain to be systematically addressed. A more comprehensive view on therapeutic options, quality of life determinants, and epilepsy socioeducation would be needed.

## 5. Conclusions

Both SSA and the global population exhibit similar underlying patterns in the prevalence of epilepsy, with higher rates observed in pediatric populations compared to adults. However, the prevalence can be significantly higher in SSA, especially for higher rates of infectious diseases, socioeconomic challenges, and limited access to healthcare services. Classification and diagnosis may be more difficult due to a lack of resources and diagnostic tools, such as EEG and neuroimaging.

Epilepsy poses a major public health burden, especially in nations with limited resources, and even more so in SSA. In this setting, infectious diseases and head injuries may play a major etiological role; however, further research is necessary to better estimate the contribution of genetic factors. Diagnosing and understanding the root cause of epilepsy is difficult due to a lack of equipment and specialists. Proper management of epilepsy hinges on precise semiotic and etiological diagnosis. However, our review reveals that in SSA, epilepsy is often poorly defined in terms of semiotic features, and etiological classification is commonly lacking. PWE also face significant social challenges due to stigma and limited access to treatment, impacting their quality of life and straining healthcare systems.

The path forward involves developing culturally sensitive diagnostic tools, increasing access to affordable medication and treatment plans, and educating the public to reduce stigma and promote social inclusion for PWE. Additionally, further research is needed to explore the unique causes of epilepsy in Africa, such as genetic and metabolic factors. Public health strategies to prevent epilepsy, like reducing complications during childbirth and head injuries, can also significantly improve the situation.

## Figures and Tables

**Table 1:** A comprehensive overview of the epidemiology of epilepsy. The table provides an overview of the prevalence and incidence rates observed in different Sub-Saharan countries. Wherever feasible, comparisons are drawn with global rates or rates from other industrialized countries that are not tropical. Furthermore, specific etiologies are referenced in the text. LMIC = low- and middle-income country; HIC = high-income country; ACE = active convulsive epilepsy.

Study	Topic and Type of Study	Main Findings
Fiest et al., 2017 [11]	International prevalence and incidence of epilepsy, systematic review and meta-analysis.	Worldwide pooled point prevalence of active epilepsy 6.38 per 1000 persons and pooled incidence rate of epilepsy 61.44 per 100,000 person-years. LMIC prevalence of active epilepsy 6.68 per 1000 persons compared to HIC prevalence of active epilepsy 5.49 per 1000 persons.
Preux et al., 2005 [12]	Review of epidemiology and etiology of epilepsy in Sub-Saharan Africa.	Annual incidences of 63–158 per 100,000 inhabitants, higher than industrialized countries in nontropical areas of 40–70 per 100,000 inhabitants. Prevalence of epilepsy variable even in the same country with a median prevalence found in door-to-door studies of 15 per 1000 people.
Beghi et al., 2020 [4]	Review of global epidemiology of epilepsy.	Incidence higher in LMICs than HICs, 139.0 vs. 48.9. As the incidence of epilepsy appears higher in most LMICs, the overlapping prevalence can be explained by misdiagnosis, acute symptomatic seizures and premature mortality.
Kaiser et al., 1998 [14]	Incidence of epilepsy in Uganda, related to onchocerciasis.	Crude incidence rate of 215 per 100,000 person-years with a significant difference between zones of high and low onchocerciasis endemicity.
Rwiza et al., 1992 [15]	Prevalence and incidence of epilepsy in a rural Tanzanian District.	Prevalence of active epilepsy 10.2 in 1000, ranging from 5.1 to 37.1 in 1000 (age-adjusted 5.8–37.0). In a 10-year period (1979–1988), annual incidence of 73.3 in 100,000.
Tekle-Haimanot et al., 1997 [16]	Incidence of Epilepsy in Rural Central Ethiopia.	Annual incidence of 64 in 100,000 inhabitants. The corresponding rate for males was 72 (CI 42–102); for females, it was 57 (CI 31–84). The highest age-specific incidence occurred in the youngest age groups (0–9 years).
Edwards et al., 2008 [17]	Prevalence and risk factors for ACE in a rural district of Kenya.	Overall prevalence of ACE 2.9 per 1000 people. On a 5 year cut off for date of last seizure, unadjusted prevalence of ACE 3.1 per 1000 (2.8–3.4 per 1000).
Garrez et al., 2024 [18]	Epilepsy prevalence in rural Southern Rwanda.	Crude, unadjusted prevalence of lifetime epilepsy 76.2 per 1000 people. Prevalence adjusted for screening sensitivity 80.1 per 1000 people when. Crude prevalence standardized to the age distribution from Rwanda 78.3 per 1000 when, 89.1 per 1000 from the United States, and 85.3 per 1000 worldwide.
Mwanga et al., 2024 [19]	Prevalence epilepsy in urban informal settlements in Kenya.	Crude prevalence of epilepsy 9.4 per 1000 people. Prevalence adjusted for attrition 11.5 per 1000 people and 11.9 per 1000 people (11.0–12.8) when adjusted for attrition and sensitivity.
Biset et al., 2024 [20]	Systematic review and meta-analysis of prevalence, incidence, and trends of epilepsy among children and adolescents in Africa.	Pooled prevalence of cumulative epilepsy was 17.3 per 1000 children. Pooled prevalence of lifetime and ACE were 18.6 and 6.8 per 1000 children respectively. Pooled prevalence of unclassified epilepsy was 45.5 per 1000 children. Pooled prevalence of epilepsy in high parasite endemic areas was 44 per 1000 children. Pooled prevalence of epilepsy in the general population was 8 per 1000 children. The highest prevalence of epilepsy was reported in Southern African countries (129.3/1000) followed by Central African countries (32.3/1000) and Northern African countries (24.1/1000).

**Table 3:** Etiology of epilepsy in Sub-Saharan Africa. The table shows an overview of the different causes of epilepsy. Structural causes and infectious etiologies appear to be the most common in the Sub-Saharan African population. ACE = acute convulsive epilepsy, CWE = children with epilepsy DEE = developmental and epileptic encephalopathies, MRI = magnetic resonance imaging, PWE = people with epilepsy, HS = hippocampal sclerosis, OAEs = onchocerciasis-associated epilepsies, Ov = Onchocerca volvulus, CNS = central nervous system.

Study	Population	Type of Study	Main Findings
Ngugi et al., 2013 [42]	South Africa, Ghana, Kenya, Tanzania, Uganda.	Observational cross-sectional, case–control.	Risk factors for active convulsive epilepsy in children (aged < 18 y) (*p* < 0.0001): seizure in the family; abnormal antenatal period; difficulties feeding, crying, or breathing; any other problems after birth.
Kamuyu et al., 2014 [45]	South Africa, Ghana, Kenya, Tanzania, Uganda.	Observational cross-sectional, case–control.	Association between individual parasites (O. volvulus, T. canis and T. gondii) and ACE prevalence. Greater combined effect for coinfection with T. gondii and O. volvulus.
Christensen et al., 2015 [46]	Malawi, Kenya, Uganda, Gabon, Mali.	Review.	Cerebral malaria is associated with an increased risk of epilepsy as a long-term adverse outcome (OR 4.68, 95% CI 2.52–8.70).
Lompo et al., 2018 [39]	Burkina Faso.	Observational cross-sectional.	Main etiologies among nongenetic epilepsies: - Neurocutaneous syndrome 1.7%. - Malformation of cortical development 2.6%. - Sequelae of cranial and brain trauma 5.6%. - Sequelae of central nervous system infection 29.6%. - Brain tumors 1.7%. - Sequelae of perinatal cerebral suffering 34.8%. - Hippocampal sclerosis 1.7%.
Samia et al., 2019 [30]	Kenya.	Review.	Common risk factors in children for both epilepsy and acute seizures included adverse perinatal events, meningitis, malaria, febrile seizures, and family history of epilepsy.
Sahlu et al., 2019 [55]	Burkina Faso.	Randomized, case–control.	Positive association between seropositivity to cysticercal antigens and active epilepsy (prevalence odds ratio: 2.40 (95% CI: 1.15–5.00)).
Egesa et al., 2022 [35]	Kenya.	Review.	The most common etiologies of epilepsy: infectious (44.8%) and structural (36.4%). Structural causes were higher in CWE (44.9%) than in adults (26.9%), (*p* < 0.001). About 24.6% of persons had undetermined epilepsy causes.
Essajee et al., 2022 [56]	South Africa.	Observational prospective.	A genetic underlying cause for DEE was identified in 18 of 41 CWE (diagnostic yield 43.9%) by performing a targeted next generation sequencing analysis. The more common pathogenic variants were found in SCN1A (n = 7), KANSL1 (n = 2), KCNQ2 (n = 2) and CDKL5 (n = 2).
Apolot et al., 2022 [54]	Uganda.	Observational cross-sectional.	The prevalence of structural abnormalities among CWE was 74.15%. Acquired structural brain abnormalities were the commonest at 69.22% with hippocampal sclerosis (HS) leading while disorders of cortical development were the most common congenital causes.
Mazumder et al., 2022 [51]	Uganda.	Observational longitudinal, case–control.	Comparison between structural changes in the brain MRI between nodding syndrome and other forms of OAE; relation between structural changes to the OV-induced immune responses and level of disability. Treatment of onchocerciasis reduces the seizure burden.
Siewe Fodjo et al., 2022 [53]	Cameroon.	Observational longitudinal.	Documented ongoing transmission of onchocerciasis alongside a suboptimal ivermectin coverage: the patients with epilepsy were more Ov-infected than participants without epilepsy, supporting the existence of an association between onchocerciasis and epilepsy. Having O. volvulus infection and especially higher microfilarial loads was significantly associated with epilepsy.
Esterhuizen et al., 2023 [57]	South Africa.	Observational longitudinal.	Tested genetically 234 naive South African children diagnosed with/possible DEE: 41 (of 234) children with likely/pathogenic variants, 26 had variants supporting precision therapy. Importance of early genetic diagnosis in DEE. We designed the “Think-Genetics” strategy for early recognition, appropriate interim management, and genetic testing for DEE in resource-constrained settings.
Bhattacharyya et al., 2023 [48]	South Sudan.	Diagnostic/prognostic.	Development of a mathematical model to quantified transmission, disease parameters and predict the impact of ivermectin mass drug administration (MDA) and vector control. The model estimated an OAE prevalence of 4.1% in Maridi County. The OAE incidence is expected to rapidly decrease by >50% within the first five years of implementing annual MDA with good coverage (≥70%). With vector control at a high efficacy level (around 80% reduction in blackfly biting rates) as the sole strategy, the reduction is slower, requiring about 10 years to halve the OAE incidence. Increasing the efficacy levels of vector control, simultaneously with MDA, yielded better results in preventing new cases of OAE.
Edridge et al., 2023 [44]	Uganda, Malawi, and Rwanda.	Observational.	Viral and bacterial CNS infections and IMDs are prevalent causes of severe acute encephalopathy in children in Uganda, Malawi, and Rwanda. These causes are likely to be missed by conventional diagnostics and are associated with poor outcome of disease.
Jada et al., 2023 [47]	Sudan.	Longitudinal, population-based.	Onchocerciasis may induce seizures through direct or indirect mechanisms: strengthening onchocerciasis elimination interventions can decrease the incidence of epilepsy, including nodding syndrome.
Kariuki et al., 2024 [38]	Kenya and South Africa.	Observational retrospective.	MRI abnormalities were found in 140 of 240 of PWE in Kenya, and in 62 of 91 in South Africa (pooled modeled prevalence = 61%) Abnormalities were common in those with a history of adverse perinatal events (65%, exposure to parasitic infections (69%) and focal electroencephalographic features (68%), 95% CI: 60%–76%). Mesial temporal sclerosis (43%) and gliosis (34%) were the most frequent abnormalities found.
Colebunders et al., 2024 [49]	Sub-Saharan Africa.	Review.	Underline the strong association between onchocerciasis and seizures, reinforcing the concept of OAE; need for case definition to estimate the burden of disease and identify onchocerciasis-endemic areas requiring intensification of elimination programs and integration of epilepsy care. To reduce OAE burden, enhance collaboration with mental health programs at community, national, and international levels is required.
Amaral et al., 2024 [52]	Sudan.	Observational prospective.	Observed decrease in epilepsy incidence despite suboptimal cumulative community-directed treatment with ivermectin coverage highlights the potential impact of onchocerciasis control efforts and underscores the need to strengthen these efforts.

## Data Availability

The data presented in this study are available on request from the corresponding author.

## Supplementary Materials

The following supporting information can be downloaded at: https://www.mdpi.com/article/10.3390/jcm13216396/s1, Details of epidemiology and diagnostic tools. References [86,87,88,89] are cited in the supplementary materials.

## Abbreviations

WHO = World Health Organization; PWE = people with epilepsy; CWE = children with epilepsy; LAMICs = low- and middle-income countries; HICs = high-income countries; SSA = Sub-Saharan Africa; ASM = antiseizure medications; NS = Nodding syndrome; ART = antiretroviral therapy; cART = combined antiretroviral therapy; DALYs = disability-adjusted life years; ILAE = International League Against Epilepsy; SMR = standardized mortality ratio; DEE = developmental and epileptic encephalopathies; NGS = next-generation sequencing; MRI = magnetic resonance imaging; CT = computerized tomography; EEG = electroencephalogram; CNS = central nervous system.
