# Supplementary material for: Diagnosis and Classification of Pediatric Epilepsy in Sub-Saharan Africa: A Comprehensive Review
Source: J Clin Med. 2024 Oct 25;13(21):6396. doi: 10.3390/jcm13216396 (PMC11545903; doi:10.3390/jcm13216396)
Supplement: Supplementary file 1 [file jcm-13-06396-s001.zip › jcm-3248538-supplementary.pdf]

Epidemiology of Epilepsy: Sub-Saharan Africa vs. HIC

Aspect	Sub-Saharan Africa	HIC
Prevalence Rates	5 to 10%	0.5 to 1%
Causes	Infectious diseases (e.g., neurocysticercosis, HIV), trauma, birth injuries	Structural brain abnormalities, genetic factors, degenerative diseases (e.g., stroke)
Age of Onset	Higher prevalence in childhood/adolescence due to preventable causes	Varied onset, often in late adolescence/early adulthood
Healthcare Access and Treatment	Limited access, stigma, and misconceptions about epilepsy	Better access to healthcare, advanced treatments, and education
Mortality and Morbidity	Higher morbidity and mortality due to lack of treatment and comorbidities	Lower mortality due to effective management and emergency care
Socioeconomic Factors	High poverty levels, low education, underfunded healthcare systems	Higher socioeconomic status allows for better health outcomes

Table providing a general overview of the differences in the epidemiology of epilepsy in different socio-economic settings

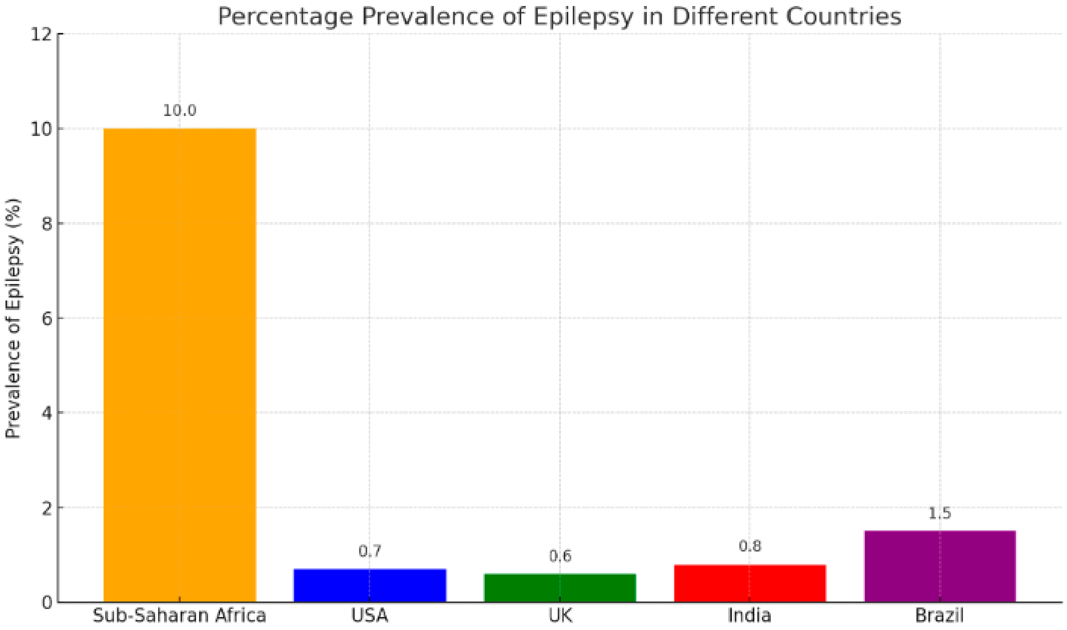

Ngugi, A. K., et al. (2010). "Prevalence of active epilepsy in sub-Saharan Africa: a systematic review." *Tropical Medicine & International Health*, 15(3), 296-304.
Hesdorffer, D. C., et al. (2011). "Incidence of unprovoked seizures and epilepsy in Rochester, Minnesota, 1980-2000." *Neurology*, 76(1), 20-25.
Kanner, A. M. (2009). "Epilepsy: an overview of the epidemiology." *Journal of Neurology, Neurosurgery & Psychiatry*, 80(2), 191-197.
Sinha, S. K., et al. (2001). "A study of prevalence of epilepsy in a rural community in India." *Epilepsia*, 42(1), 46-49.
Cendes, F., et al. (2005). "Prevalence of epilepsy in Brazil: a population-based study." *Neurology*, 64(3), 559-564.

Regarding the diagnostic tools developed and validated in the African population for the investigation of epilepsy, in the selected 10 years' time frame, 5 articles were found. 3 articles describing the development and validation of diagnostic questionnaires, 1 article assessing the impact of screening tools in low-resource countries, 1 article investigating and describing the access to electrophysiology services. **Patel et al.** proposed a diagnostic questionnaire validated on the pediatric population (6 moths to 18 years), in comparison with the other authors that validated the questionnaire from 6 years of age. **Vergonjeanne et al.** described the two decades experience of use of the questionnaire for Investigation of Epilepsy in Tropical Countries (IENT). The IENT questionnaire includes 9 sections ranging from anamnestic data to clinical examination, including a section on etiological investigations and treatment. The first two sections, "demographic data" and "screening" can be filled by a non-medical investigator. All three studies used a two-step approach with a first step characterized by screening participants randomly selected or identified via door-to-door visits, with a few screening questions and the administration of a questionnaire by non-medical healthcare workers. In step two, all individuals screened positive from the questionnaire were invited to be examined by a neurologist. **Jones et al.** developed a tool that, although limited to convulsive seizures, has the aim to direct the individual to see the most appropriate clinician and to keep track of the patient through the application.

According to **Shalu et al** prevalence estimates obtained from the two-stage approach may be seriously biased due to the failure to consider the imperfect validity of the tests, used in the first or second stage, or in both stages, leading to verification bias and misclassification errors. To overcome biases, it is important to compare the collected data with statistically adjusted one, obtained according to statistical analysis such as the Bayesian latent-class models.

In the last study, **Kander et al.,** investigate the access and the level of competence of the practitioner of electrophysiology services in sub-Saharan Africa (SSA), with a special focus on pediatric electrophysiology. As gold standard, EEG study performed by a neurophysiology technologist and interpreted by a specialist with formal training in epileptology, can assist and enhance the diagnosis, delineation of syndromes and therefore the management of epilepsy. According to the study, in terms of paediatric EEGs, the survey could not address evidence for improved care, nevertheless, most participants agreed that a viable training model would be beneficial to improve diagnosis and management for pediatric patients.

Article	Population	Type of Study	Intervention	Indicator of accuracy	Outcome	Positive Aspects	Limitation
**Jones et al. 2023**	Sub-Saharan Africa; over 6 years	Case-controls study	Questionnaire for Community-based health-care workers. 8 binary questions	Sensitivity, specificity, and positive and negative predictive values were 97·5% (93·7–99·3), 82·4% (71·2–90·5), 92·9% (87·9–96·3), and 93·3% (83·8–98·2; table 2).	Diagnosis of convulsive epilepsy	Free app Epilepsy Diagnostic Companion (EDC); facilitating more appropriate onward referral to a neurologist	Convulsive seizures only; Regional limits; difficulties in follow-up participation; selection bias; prevalence bias;
**Patel et al. 2016**	Tanzania and Zambia; 6 months to 18 years	Observational study	To patient's caregiver by a nonmedical staff member. 15 most discriminating features of semiology characteristics	Sensitivity of 78% and positive predictive value of 81.5%	Discriminate focal from generalized seizures	Translated into local dialects.	Poor for translation (difficulties with precise classification of specific seizure semiologies); screening questions do not include all types of epilepsy; does not include questions on concomitant illness and therefore possible etiologies
**Vergonjeanne et al. 2021**	African population; all ages.	Observational study	Questionnaire for investigation of epilepsy in tropical countries (IENT questionnaire). 9 sections with a total of 213 items combining both binary and open answer.<br>The first two sections can be filled by a non-medical investigator.	The sensitivity and specificity were estimated to be 95.1% (95% CI: 87.3%–98.4%) and 65.6% (95% CI: 57.5%–72.9%), respectively.	To screen (estimate the prevalence in an area); to identify clinical forms of epilepsy; to determine etiologies; to describe treatment	Available in several languages, such as French, English, Spanish, and Portuguese.	Validated in 2000, need an update according to the new classification of epilepsy; a semiological support could be added, in order to improve the classification of seizures and epilepsy.

Development and validation of a diagnostic aid for convulsive epilepsy in sub-Saharan Africa: a retrospective case-control study.

- Convulsive epilepsy
- sub-Saharan African population
- Case-controls study
- Method: two screening questions during the routine, door-to-door census to heads of households about convulsions in each individual at the dwelling; second stage more detailed questionnaire was administered by field workers to individuals who screened positive in the first stage; the third stage, people positive at the second stage were assessed by clinicians who made a final diagnosis of epilepsy: each participant was phenotyped according to: clinical history; clinical examination; seizure description; and electroencephalogram (EEG) interpretation.
- The primary outcome was a diagnosis of convulsive epilepsy with an EEG-aided diagnosis confirmed by a neurologist specialised in epilepsy. Controls were people without a diagnosis of convulsive epilepsy who completed the same clinical pathway.
- 8 binary questions
- Community-based health-care workers
- Older than 6 years of age
- Free app, the Epilepsy Diagnostic Companion (EDC)
- Individual triage by non-physician health-care workers and facilitating more appropriate onward referral to a neurologist for diagnostic confirmation of convulsive epilepsy
- Translated into the languages represented at each of the study sites
- CONS: logistical difficulties in contacting eligible follow-up participants; prevalence estimates from these studies may not reflect other regions of sub-Saharan Africa given that the study sites were selected on the criteria of endemicity of potential risk factors and the availability of minimum resources required to support the studies; selection bias, and prevalence bias.

A pediatric epilepsy diagnostic tool for use in resource-limited settings: A pilot study

- Questionnaire to discriminate focal from generalized seizures
- Three initial screening questions from existing validated questionnaires were also included to confirm that the child had met criteria for epilepsy (confirming impairment of consciousness, recurrent events, and events without fever; simple motor seizures were excluded)
- 15 most discriminating features of semiology characteristics distinguishing between focal and generalized seizures in children formatted into yes/no questions.
- Translated into local dialects
- Administered to patient's caregiver by a nonmedical staff member
- 6 months to 18 years
- Methods: administration of the questionnaire to patient's caregiver by a nonmedical staff member, were given a diagnosis of focal or generalized epilepsy by questionnaire, and by combined electroclinical diagnosis (clinical evaluation and EEG). In cases of dis- crepancy between EEG interpretations, a consensus diagnosis was reached between 2 board-certified pediatric neurologists with addition- al certification in clinical neurophysiology
- Limitations: poor for translation (difficulties with precise classification of specific seizure semiologies es. myoclonic seizures did not translate well in Tanzania or Zambia); screening questions do not include all types of epilepsy (es. simple motor seizures are not associated with an impairment of consciousness and, therefore, by design, are excluded from the questionnaire based on the three initial screening questions). The questionnaire does not include questions on concomitant illness and therefore possible etiologies (es in these regions, often prolonged seizures or repeated seizures in the setting of illness may be treated as epilepsy because of known subsequent elevated risk for seizures).

- Questionnaires are inherently limited in diagnostic accuracy by omitting subtlety and detail, and are not intended to replace a specialist but rather provide diagnostic support in regions where no specialty care is available.

Epidemiology of Epilepsy in Low- and Middle- Income Countries: Experience of a Standardized Questionnaire over the Past Two Decades

- Questionnaire for investigation of epilepsy in tropical countries (IENT questionnaire)
- Following guidelines of the International League Against Epilepsy (ILAE)
- 9 sections: "demographic data" (21 questions, 3 are optional), "screening" (5 questions + 1 if af least one answer is yes), "confirmation of diagnosis" (6 questions), "natural history of the seizure disorder" (30 questions), "past medical history" (49 questions), "clinical examination" (19 questions, including any abnormalities at the physical examination), "paraclinical examinations"(45 items to asses), "etiology" (4 questions) and "treatment. (31 questions, including treatment follow-up)"
- Binary and open answer.
- The first two sections can be filled by a non medical investigator however it is important that all field-investigators should be trained and have a working knowledge of the different seizure type. If one of the answer is positive, the investigator must go in filling in the questionnaire and the subject must be examined by a physician.
- The section Confirmation must be filled by a physician.
- Clinical examination: general state of health of the subject must be assessed, define ad poor if there is a loss of weight with asthenia, and difficulties in daily activities, average if there is asthenia or loss of weight but no problem in daily activities, good if there is no weight loss and no asthenia.
- Paraclinical examinations: relatively complex and optional (it should not resctrict the use of the questionnaire. 5 parts: blood investigations, neuro-imaging techniques, EEG, serologies and microbiology.
- Etiology: idiopathic, syntomatic, cryptogenic.
- The IENT questionnaire meets 4 objectives: (i) to screen (using the first three section you can estimate the prevalence in an area), (ii) to identify clinical forms of epilepsy (using the first 4 sections), (iii) to determine etiologies, and (iv) to describe treatment. In 2011, a first review of the use was done, but without updating the tool.
- METHOD: investigators asked the 5 screening questions. If at least one answer was "yes," the subject was a "suspect" ($n = 131$). All suspect subjects were re-examined by the neurologist, and the diagnosis of epilepsy was confirmed for 82 subjects [99]. The sensitivity and specificity were estimated to be 95.1% (95% CI: 87.3%–98.4%) and 65.6% (95% CI: 57.5%–72.9%), respectively.
- Used alone, the screening section is rapidly applied.
- The questionnaire is available in several languages, such as French, English, Spanish, and Portuguese.
- Validated in 2000, need an update The IENT questionnaire needs to be updated ac- cording to the new classification of epilepsy. In addition, a semiological support could be added, in order to im- prove the classification of seizures and epilepsy for health workers in primary care.
-

The impact of imperfect screening tools on measuring the prevalence of epilepsy and headaches in Burkina Faso

- Baseline screening questionnaire followed by neurological examination data from a cluster randomized controlled trial
- 5 Questions related to epilepsy were based on the International League Against Epilepsy screening of epilepsy questionnaire developed by Preux *et al (IENT)*
- from 6 years
- Despite its frequent use, the prevalence estimates from the two-step approach can be seriously biased from failure to account for the imperfect validity of the tests, referred to as misclassification error hereinafter, employed either at step one or step two, or both.
- In step two, all individuals screened positive for either epilepsy or WSCH from the screening questionnaire were invited to be examined by a study physician. In addition, 231 screenednegative

individuals were randomly selected to be examined by the physician. The medical examination results were collected on a medical examination questionnaire.

Understanding the landscape of electrophysiology services for children in sub-Saharan Africa

Incidence of epilepsy is greatest in infancy and childhood; this is especially evident in sub-Saharan Africa (SSA). The aim of this study was to understand access to electrophysiology services in SSA including which health practitioner performs and interprets paediatric electroencephalogram (EEG) studies as well as their training in paediatric EEG. A web-based survey was sent to a cohort of health care practitioners who manage children with epilepsy in SSA. The questions addressed whether EEG was available to these health care practitioners, how the practitioners accessed EEG and who assisted interpretation of the study results. The survey was circulated (June-December 2019) to 305 participants from 32 African countries. A total of 73 (16 partial and 57 complete) surveys were returned from 18 countries. The respondents fell into two main categories: those with access to an EEG machine (44/73; 60%) and those without access to an EEG machine (29/73; 40%). For 32% (23/73), there was no dedicated technician and for 34% (25/73) no neurologist. Access to a neurologist resulted in the highest proportion of EEGs performed per annum. Of the respondents, 77% (56/73) agreed that there was a need for a paediatric apprenticeship in EEG skills. Qualitative data to justify need for paediatric EEG training was grouped into three themes: (1) "professional development"; (2) "better care"; and (3) "help paediatric patients and neurologists". There is a lack of paediatric EEG training amongst doctors and technicians working with epilepsy in SSA. Expanding training beyond current capacity in SSA, for technicians and practitioners involved in EEG, is necessary.

- Understand access to electrophysiology services in SSA including which health practitioner performs and interprets paediatric electroencephalogram (EEG) studies as well as their training in paediatric EEG.
- Whilst epilepsy is diagnosed on clinical grounds, performing an electroenceph- alogram (EEG), in conjunction with the clinical assessment, can assist and en- hance the diagnosis, delineation of syndromes and the management of epilepsy. As gold standard, an EEG study should be performed by a neurophysiology technologist or EEG technician and interpreted by a specialist with formal training in epileptology.
- 15-minute web-based survey consisting of 42 questions for health care practitioners who manage children with epilepsy - how the health care practitioners were able to access care for their patients with paediatric neurology conditions inclusive of how this related to EEG services in their country, with a special focus on paediatric electrophysiology.
- data on access to a neurologist, waiting times for EEG, number of EEG studies performed, personnel performing EEGs, their training experience (formal or informal), the type of practition- er interpreting paediatric studies and the usefulness of an apprenticeship in paediatric EEGs. The questions were mainly drop-down box options with a few open- ended questions, formatted to provide specific qualitative data for analysis. The aim of the study was to investigate and understand the extent and nature of access to electroencephalography studies in SSA, especially for paediatric patients. Also, the level of competence of the practitioner performing and interpreting the studies. In addition, we looked at an apprenticeship training programme that will focus on technicians to learn basic paediatric EEG interpretation for safe practice.
- Access to a neurologist was also more likely in a tertiary (urban) than rural setting. Most children with epilepsy are treated by medical officers, paediatricians and psychiatrists, rather than specialist child neurologists. Access to an EEG service was available in a higher proportion than expected but this was when there was a neurologist available. A neurologist was statistically more likely to undertake more EEGs per year compared to services that lacked access to a neurologist. Paediatric neurologists requested more appropriate EEGs compared to paediatricians and non-specialists. However, the survey did not capture how often the EEG studies resulted in a change in practice, neverthe- less, this trend was also illustrated in another study by the authors. However, in terms of paediatric EEGs, the

survey could not address evidence for improved care, nevertheless, the verbatim quotes supported why paediatric apprenticeship training would be beneficial.

- District hospitals also tend not to be equipped with specialised equipment and many patients must travel long distances in order to be tested, often at a significant service fee.
- The overall response for why apprenticeship training was needed was to improve diagnosis and management for paediatric patients as well as improve skills both for doctors and technicians.
- Viable training models are needed to target the deficit of practitioners skilled in paediatric EEG in SSA.

EXTRA_Diagnosis of epileptic seizures by community health workers using a mobile app: A comparison with physicians and a neurologist

- App on a tablet computer
- India and Nepal
- non- physician health workers (NPHWs)
- determination about whether episodes of loss or alteration of consciousness are epileptic or non-epileptic in nature, and a tool in the form of a phone app for this purpose has been developed in Nepal [11]. This was derived using Bayesian principles by starting with the pre-test probability of episodes being epileptic and then calculating the likelihood ratios (LRs) of episodes being epileptic for each of 40 variables. The eventual algorithm asks the most eloquent 11 questions (those with LR > 3) about the episodes (Table 1), weights the answers based on the LR, and arrives at a probability score of the episodes being epileptic or not.
- In accordance with the validation study app scores of 80 or greater were recorded as epileptic and below 31 as non- epileptic with the rest being uncertain.
- All identified patients were referred to a local doctor for further diagnosis and management. The doctor had no access to the app score.
- The patient was then seen by a neurologist (MBS) at her next visit
- If epilepsy was possible then predetermined questions were asked about the nature of the episodes. These questions were grouped around broad themes: demography (11 questions), events preceding the episode, eyewitness description of the episode when available, events following the episode and predisposing factors (33 questions). If the patient had already been seen before other 6 questions on preceding examination, CT, MRI, EEG and drug usage.
- 11 demographic questions
- 33 questions on the

1. Name
2. Date
3. Clinic
4. Clinic number
5. Age
6. Sex
7. Village
8. Mobile
9. Occupation
10. Travel time to clinic in hours
11. Can read Nepali
12. Is this your first attendance for these blackouts?
13. Age at first attack
14. How many attacks?
15. How many in last month?
16. How many days since attack?
17. Time of attacks
18. Attacks lying down

19. Precipitating factors
20. Any warning?
21. How many minutes for?
22. Do these ever occur without an attack?
23. How many in last month?
24. Eyewitness present?
25. What happens first?
26. Any colour change?
27. Any stiffness?
28. Any shaking?
29. Total minutes of abnormal movement
30. Are eyes open or closed?
31. Can you communicate when stiff/shaking?
32. After stiffness/shaking stops how long before starts to come round?
33. Total minutes till normal
34. Does head turn to right or left?
35. Any incontinence?
36. Any tongue biting?
37. Are you sleepy or confused afterwards?
38. Are your muscles sore?
39. Is one arm or leg weak afterwards?
40. If so which?
41. Predisposing factors
42. If Family history how many affected?
43. Other illnesses
44. Other drugs

IF SEEN BEFORE,

45. Year first seen
46. EEG
47. CT
48. MRI
49. Present drug
50. Dose
